# Comparative Analysis of Mitochondrial Genome from *Phormictopus cancerides* (Arachnida: Theraphosidae) with Phylogenetic Implications

**DOI:** 10.3390/cimb47060448

**Published:** 2025-06-11

**Authors:** Hongjian Chen, Wei Xu, Hongyi Liu, Yiwen Yang, Ming Gao

**Affiliations:** 1 School of Landscape and Horticulture, Yangzhou Polytechnic College, Yangzhou 225009, China; 2 College of Forestry, Nanjing Forestry University, Nanjing 210037, China; yangyiwen@njfu.edu.cn; 3 College of Life Sciences, Nanjing Forestry University, Nanjing 210037, China; xuwei2001@njfu.edu.cn (W.X.); hongyi_liu@njfu.edu.cn (H.L.); gming2876@163.com (M.G.)

**Keywords:** Theraphosidae, mitochondrial genome, *Phormictopus cancerides*, tarantulas

## Abstract

Tarantulas represent a highly diverse taxonomic group and play a crucial role in ecosystems. To gain a deeper understanding of the evolutionary relationships within the family Theraphosidae, in this study, we characterized the mitochondrial genome (mitogenome) of *Phormictopus cancerides* for the first time. The mitogenome is a typical circular double-stranded molecule, with a size of 13,776 bp. *P. cancerides* exhibited an A/T nucleotide preference (61.9–68.5% A + T content), with their rRNAs and tRNAs showing higher values than PCGs and the CR. The genes and the gene order were consistent with other Theraphosidae mitogenomes. The mitogenome was compacted and showed a bias for A/T. Ka/Ks analyses showed that the *ND3* gene had the highest evolutionary rate, while the *COX1* gene displayed a relatively slower evolution. Our phylogenetic analysis based on mitogenomes showed the subfamily Theraphosinae is closely related to the subfamily Harpactirinae and the subfamily Selenocosmiinae. Our results could contribute to the study of relationships within the family Theraphosidae and lay the foundation for further studies on tarantulas.

## 1. Introduction

Theraphosidae, a family within the class Arachnida, are commonly referred to as tarantulas [1]. To date, the family Theraphosidae encompasses 156 described genera, with over 1039 extant species [2], showcasing an extraordinary degree of biodiversity. Tarantulas are predators in their respective ecosystems, fulfilling a vital ecological function in their native habitats. Firstly, they contribute to the natural regulation of prey populations. By preying on a variety of small organisms, they help maintain the balance of the food web, ensuring that certain prey species do not overpopulate and cause disruptions to the ecosystem. Secondly, tarantulas can act as bioindicators when evaluating soil health [3,4]. Their presence, abundance, and distribution can reflect the quality of the soil environment, providing valuable insights into the overall ecological status of an area. However, wild populations of tarantulas are facing intense capture pressure [5]. They are highly sought after in the international pet trade, with many being sold abroad. This excessive exploitation could lead to a significant decline in their wild populations and even result in local extinctions. Given these threats, it is of utmost importance to increase our focus on the conservation and study of these species to safeguard their survival and maintain the ecological balance they support.

Despite their ecological importance and diverse species, research on tarantulas, especially at the molecular level, is still limited [5]. Mitochondrial genomes (mitogenomes) have emerged as powerful tools in various biological studies, including in taxonomy, population genetics, and phylogenetic analyses [6,7]. This is attributed to their distinct characteristics, including their small size, simple structure, maternal inheritance pattern, and conserved gene composition [8]. In arthropods, the mitogenome typically exists as a compact circular DNA molecule, ranging from 14 to 19 kb in size. It consists of 13 protein-coding genes (PCGs), 22 transfer RNA genes (tRNAs), 2 ribosomal RNA genes (rRNAs), and a non-coding control region (CR) [9,10]. Generally, the gene arrangement in arthropod mitogenomes is relatively conserved. However, certain invertebrate lineages, including some within the arachnid group, exhibit significant rearrangements in their mitochondrial gene order [11,12]. Understanding these gene arrangements and variations in tarantula mitogenomes could provide crucial insights into their evolutionary history, genetic diversity, and population structure. Given the current paucity of molecular research on tarantulas, investigations into their mitogenomes are particularly valuable for filling these knowledge gaps.

As of now, only three verified complete mitogenomes of the family Theraphosidae (*Brachypelma albiceps*, OK298473.1; *Cyriopagopus hainanus*, MN877932.1; *Ornithoctonus huwena*, AY309259.1) have been published in GenBank [13,14]. Partial mitochondrial sequences provide only limited information and lack detailed data regarding gene rearrangements, genetic code variations, the regulatory patterns of replication and transcription [15]. In contrast, complete mitochondrial genome sequences can offer higher resolution and sensitivity for research into evolutionary relationships. Because of the development of sequencing technology, researchers have been able to obtain sequences in large quantities [16]. Here, we sequenced the newly complete mitogenome of *Phormictopus cancerides*, a popular pet spider. We performed comparative analyses of nucleotide sequences, base compositions, and codon use and a phylogenetic analysis. This study could complement the genome and contribute to the phylogenetic analysis of tarantulas.

## 2. Materials and Methods

### 2.1. Sample Collection and DNA Extraction

The sample utilized in this study was sourced from insect markets in Zhenjiang, Jiangsu, China. Initially, the specimens collected were morphologically identified with reference to the Global Biodiversity Information Facility (GBIF, accessible at https://www.gbif.org/, accessed on 1 April 2025). The specimen collection process was carefully examined and approved by Nanjing Forestry University, guaranteeing strict adherence to relevant Chinese regulations. After collection, the specimens were kept in the Zoology Laboratory of Nanjing Forestry University. Total DNA was extracted from their shed skins using the FastPure Cell/Tissue DNA Isolation Mini Kit (produced by Vazyme™, Nanjing, China). Once the extraction was complete, the DNA sample was stored at −20 °C for future analysis.

### 2.2. Next-Generation Sequencing

Library construction and sequencing were conducted by Shanghai Personal Biotechnology Co., Ltd. (Shanghai, China) using the NovaSeq X Plus platform (Illumina, San Diego, CA, USA), which generated 150 bp paired-end reads.

To guarantee the production of high-quality data, low-quality sequences were filtered out. The quality filtering process employed a sliding-window approach to quality assessment. The sliding window was set to a size of 9 bp and a step size of 1 bp. As the window moved one base at a time, the average Q-value of the 9 bases within the window was calculated. When the average Q-value of a window fell below 2, only the second-last base and all the bases before it in that window were retained.

The clean reads obtained after this filtering process were then used to assemble the complete mitogenome. This assembly was achieved using Geneious Prime 2024 software, with the mitogenome of *B. albiceps* as the reference template. The assembly was performed under the medium sensitivity/speed setting. Consensus sequences were generated with a base call threshold of 50%, which finally led to the successful acquisition of the complete mitogenome.

### 2.3. Annotation and Sequence Analysis

Conservative domains within the mitogenome were identified through the utilization of BLAST CD-Search and the MITOS server [17,18]. The gene map of the mitogenome was generated by means of the CG View server [19]. Nucleotide bias was quantified using the formulas “AT-skew = (A − T)/(A + T)” and “GC-skew = (G − C)/(G + C)” [20]. Analyses of relative synonymous codon usage (RSCU), as well as non-synonymous (Ka) and synonymous substitutions (Ks), were performed using MEGA 12 software [21]. Images of RSCU were output via PhyloSuite v1.2.3 [22].

### 2.4. Phylogenetic Analysis

For the phylogenetic analyses, ten species belonging to the family Theraphosidae were selected. Additionally, *Asemonea sichuanensis* from Araneae-Salticidae was included as an outgroup taxon (Table 1). Sequence alignment was carried out using MAFFT v7.505 [23], and subsequent model prediction was performed with ModelFinder v2.2.0 [24]. Phylogenetic analyses of each dataset were conducted using two methods: Bayesian inference (BI) and maximum likelihood (ML). Both methods are accessible within PhyloSuite v1.2.3 [22].

Based on the Bayesian Information Criterion (BIC), the most suitable model for BI and ML was determined to be GTR + F + I + G4. The BI tree was reconstructed using MrBayes v3.2.7a [25]. Markov chains were run for one million generations, with sampling occurring every hundred generations. To obtain a consensus tree based on the majority rule, the results of duplicated analyses were combined and the first 25% of generations were discarded. The ML tree was reconstructed using IQ-TREE v2.2.0 with 5000 bootstrap replicates [26]. Finally, the phylogenetic tree was visualized and edited using the iTOL server [27].

## 3. Results

### 3.1. Mitochondrial Genome Organization

**Figure 1:**
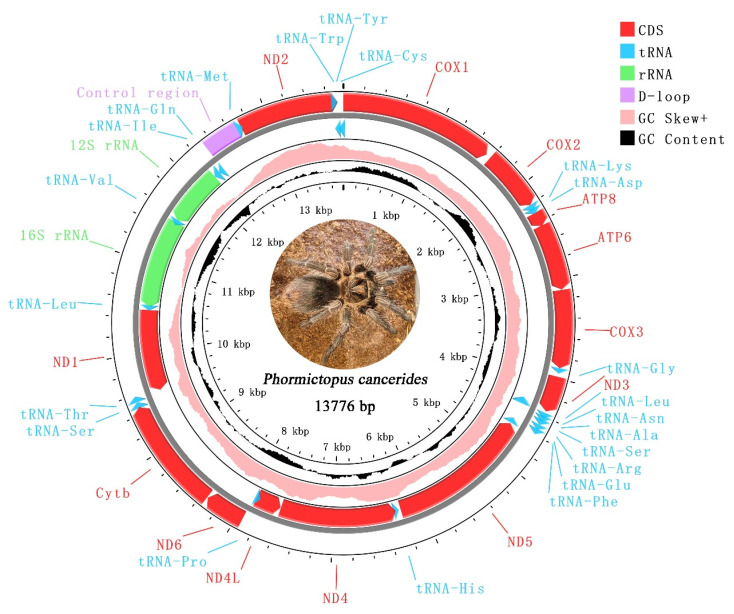
Mitochondrial genome of *Phormictopus cancerides*. The red represents PCGs; blue represents tRNAs; green represents rRNAs; purple represents CR; pink represents positive GC skew; and black represents GC content.

The complete mitogenome of *P. cancerides* presented as a typical circular double-stranded DNA molecule, with a size of 13,776 bp (Figure 1). This mitogenome contained the typical 37 mitochondrial genes (13 PCGs, 22 tRNAs, and 2 rRNAs) and 1 CR. Ten tRNAs, two rRNAs, and four PCGs were encoded on the minor strand, while the remaining genes were located on the major strand (Table 2).

The mitogenome under study was highly compact, with 24 instances of gene overlap identified. Among these, the longest gene overlap, spanning 65 bp, occurred between ND2 and tRNA-Trp (Table 2).

**Figure 2:**
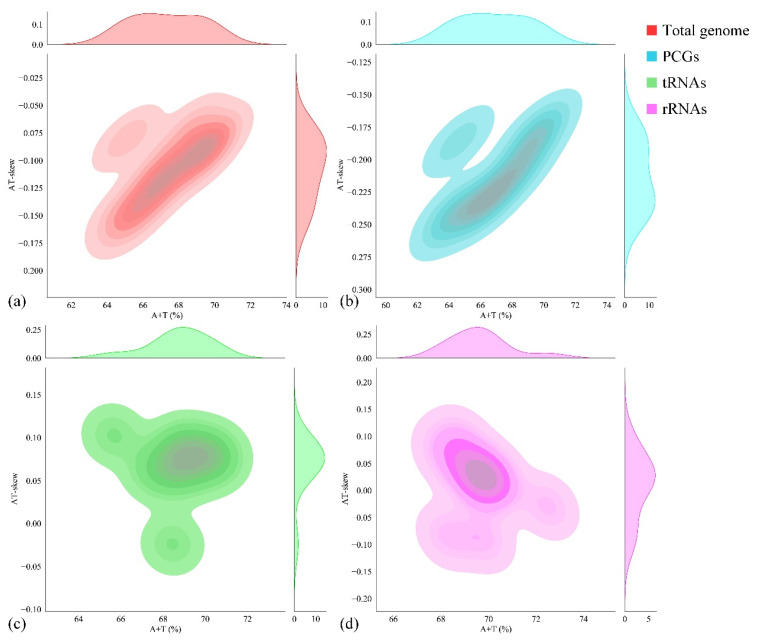
Nucleotide composition (AT skew vs. A + T content) of ten Theraphosidae mitogenomes: whole genome (**a**), PCG (**b**), tRNA (**c**), and rRNAs (**d**).

For the nucleotide composition analysis of the entire mitogenome, parameters such as A + T content, G + C content, AT skew, and GC skew were calculated (Table 3). The nucleotide composition of *P. cancerides* demonstrated a preference for A/T, with the A + T content ranging from 61.90% to 68.45%. Specifically, the rRNA and tRNA sequences had a relatively higher A + T content, while the A + T content in the PCGs and the CR was comparable to that of the entire mitogenome. Across the mitogenome, the AT skews were slightly negative, ranging from −0.186 to −0.024, and the GC skews were positive, within the range of 0.154–0.433 (Table 3). To explore the nucleotide composition among our sampled representatives of the family Theraphosidae, the A + T content and AT skew were computed for ten Theraphosidae mitogenomes. These mitogenomes belonged to four subfamilies: Theraphosinae, Harpactirinae, Ornithoctoninae, and Selenocosmiinae (Figure 2). The nucleotide composition of these ten Theraphosidae mitogenomes was found to be relatively consistent. In all cases, across the total genome, PCGs, tRNAs, and rRNAs, the A + T content was higher than the G + C content. In the total genome, the AT skews were predominantly negative, indicating that T generally occurred more frequently than A. Notably, the ratio of AT skew to AT content was similar for both the total genome and PCGs (Figure 2).

### 3.2. Protein-Coding Genes and Codon Usage

The size of all 13 PCGs was 10,782 bp, which accounted for 78.27% of the complete mitogenome. The A + T content of the PCGs was 64.60%.

The 13 inferred PCGs begin with the initiation codon ATN (ATG, ATA, or ATT) (Table 2). There are three types of termination codons (TAA, TAG, and T) found in *P. cancerides*. The frequency of the termination codon T was consistently lower than that of the other two termination codons.

**Figure 3:**
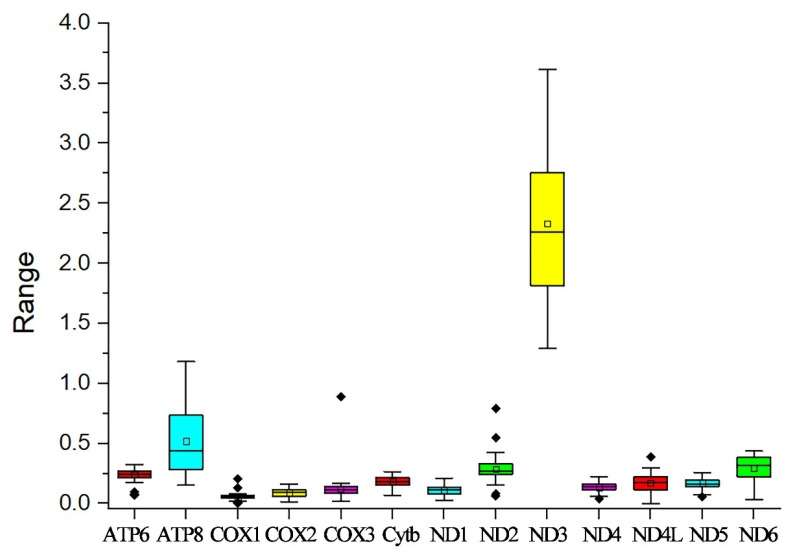
Ka/Ks values for the 13 PCGs of the ten Theraphosidae mitogenomes in this study. Black dots represent outliers.

**Figure 4:**
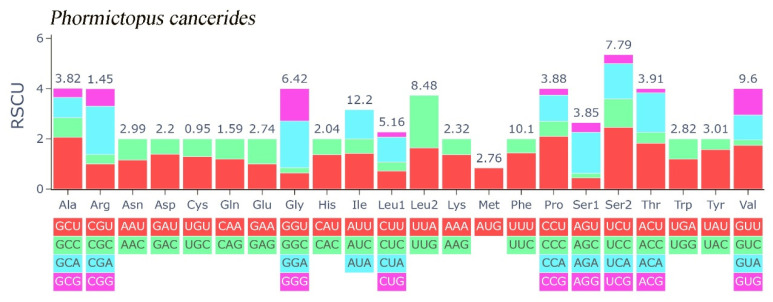
Relative synonymous codon usage of *Phormictopus cancerides* mitogenome; the stop codon is not included.

In this study, the evolutionary pathway of the PCGs was explored through analysis of the Ka/Ks ratios (Figure 3). With the exception of ND3, the Ka/Ks ratios for the majority of PCGs were determined to be less than 1. Among these PCGs, *COX1* displayed the lowest Ka/Ks value, while *ND3* had the highest Ka/Ks value. Subsequently, an RSCU analysis was carried out to investigate the codon usage patterns across the entire mitogenome (Figure 4). The findings indicated a preference for A/T in the third codon position (excluding Met), which is consistent with the observed bias toward the usage of A + T nucleotides in the PCGs.

### 3.3. Transfer RNA, Ribosomal RNA Genes, and Control Region

There were 22 tRNAs in the mitogenome. The total size of the tRNAs was 1290 bp, which accounted for 9.36% of the genome. The sizes of these tRNA genes ranged from 48 bp (*tRNA-Cys*) to 77 bp (*tRNA-Leu2*).

Two rRNA genes, namely *12S rRNA* and *16S rRNA*, were transcribed from the minor strand (Table 2). The larger rRNA (*16S rRNA*) was positioned between *tRNA-Leu1* and *tRNA-Val*, while the smaller rRNA (*12S rRNA*) was located between *tRNA-Val* and *tRNA-Ile*. The size of *12S rRNA* was 652 bp, while *16S rRNA* was 1008 bp, in the mitogenome.

One CR was found between *tRNA-Gln* and *tRNA-Met* in the new mitogenome. The size of the CR was 353 bp, accounting for 2.56% of the genome. The A + T content of the CR was 61.90%.

### 3.4. Phylogenetic Analyses

A total of ten mitogenomes from four subfamilies of the family Theraphosidae were included in our phylogenetic analyses (Table 1). Additionally, *A. sichuanensis* was selected as the outgroup to establish their phylogenetic tree. Phylogenetic trees of the BI and ML analyses were constructed based on 13 PCG nucleotide sequences; the BI and ML trees shared an identical topological structure.

**Figure 5:**
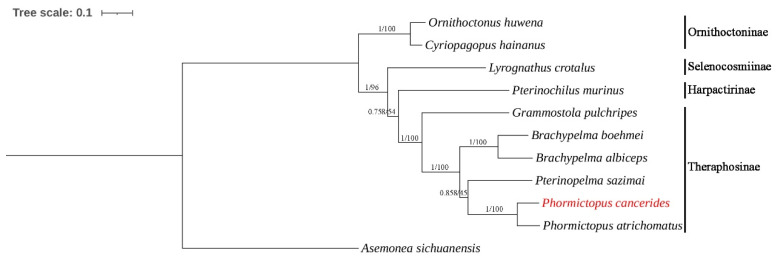
Phylogenetic tree of ten Theraphosidae species based on 13 PCGs using the BI and ML methods. *Asemonea sichuanensis* was used as the outgroup. Numbers at the nodes are statistical support values for BI (posterior probabilities)/ML (bootstrap values).

Our study presents a relatively comprehensive phylogenetic analysis of the family Theraphosidae based on mitogenomes (Figure 5). This analysis is not limited to only some of the published mitogenomes or morphological analyses. Phylogenetic analyses strongly corroborated the taxonomic relationships within the family Theraphosidae. Species belonging to the same genus or subfamily consistently grouped together to form a distinct cluster. This result showed that the subfamily Ornithoctoninae was split from other tarantulas first.

## 4. Discussion

The gene arrangement of the *P. cancerides* mitogenome is in accordance with previously reported arrangements, following the order *COX1* → *COX2* → *tRNA-Lys* → *tRNA-Asp* → *ATP8* → *ATP6* → *COX3* → *tRNA-Gly* → *ND3* → *tRNA-Leu2* → *tRNA-Asn* → *tRNA-Ala* → *tRNA-Ser1* → *tRNA-Arg* → *tRNA-Glu* → *tRNA-Phe* → *ND5* → *tRNA-His* → *ND4* → *ND4L* → *tRNA-Pro* → *ND6* → *Cytb* → *tRNA-Ser2* → *tRNA-Thr* → *ND1* → *tRNA-Leu1* → *16S rRNA* → *tRNA-Val* → *12S rRNA* → *tRNA-Ile* → *tRNA-Gln* → CR → *tRNA-Met* → *ND2* → *tRNA-Trp* → *tRNA-Tyr* → *tRNA-Cys* [13,14]. This mitogenome had a high A + T content, which was consistent with what had been observed in other tarantula species, with all values exceeding 60% [14,28].

The Ka/Ks ratios of most PCGs were found to be lower than 1. This outcome suggests that purifying selection is probably the major driving force in shaping the evolutionary patterns of the PCGs. Under ordinary conditions, selection mechanisms act to get rid of deleterious mutations, thereby keeping the protein structure and function stable [29]. *COX1* had the lowest Ka/Ks value. This implies that *COX1* has experienced intense selective pressure, causing it to evolve at a relatively slow rate [30]. *ND3* had the highest Ka/Ks value, meaning there is a relatively high rate of non-synonymous substitutions in its coding sequence. All 13 PCGs of *P. cancerides* began with ATG, ATA, or ATT start codons, whereas its congener *P. atrichomatus* used TTG as the start codon for *COX2*, *COX3*, *ND6*, and *Cytb* [31].

Not all tRNAs could be detected using tRNAscan-SE. This is highly likely to be due to the distinctive secondary structure of spider tRNAs. A significant portion of tRNAs in spiders are remarkably shortened and lack the typical dihydrouridine or TΨC arms [32].

In previous morphological studies, the subfamily Ornithoctoninae was the first to be separated, while the subfamilies Theraphosinae, Harpactirinae, and Selenocosmiinae were found to be closely related [33]. This is consistent with the findings of our present study, which is based on the 13 PCGs of their mitogenomes (Figure 5). However, for the highly diverse family Theraphosidae, the current data on complete mitogenomes remain insufficient. More molecular data are needed to conduct further analyses of the relationships in this family.

## Figures and Tables

**Table 1:** Information on the mitogenomes analyzed in this study.

Family	Subfamily	Species	GenBank ID	Size (bp)
Theraphosidae	Theraphosinae	*Brachypelma albiceps*	OK298473.1	13,856
		*Brachypelma boehmei*	OK105082.1	13,852
		*Grammostola pulchripes*	PP718135.1	14,011
		*Phormictopus atrichomatus*	PP718136.1	13,822
		*Phormictopus cancerides*	PV072888	13,776
		*Pterinopelma sazimai*	PP810236.1	13,856
	Harpactirinae	*Pterinochilus murinus*	PP718137.1	13,865
	Ornithoctoninae	*Cyriopagopus hainanus*	MN877932.1	13,874
		*Ornithoctonus huwena*	AY309259.1	13,874
	Selenocosmiinae	*Lyrognathus crotalus*	MN072398.1	13,866
Salticidae	Asemoneinae	*Asemonea sichuanensis*	MN651970.1	15,419

**Table 2:** Annotation of the *Phormictopus cancerides* mitochondrial genome.

Gene	Position		Size (bp)	Orientation	Codon		Intergenic Nucleotides (bp)
	From	To			Start	Stop	
*COX1*	1	1545	1545	+	ATA	TAG	−14
*COX2*	1594	2211	618	+	ATG	TAG	48
*tRNA-Lys*	2211	2270	60	+			−1
*tRNA-Asp*	2254	2310	57	+			−17
*ATP8*	2301	2450	150	+	ATT	TAA	−10
*ATP6*	2444	3112	669	+	ATG	TAG	−7
*COX3*	3116	3898	783	+	ATG	TAG	3
*tRNA-Gly*	3898	3952	55	+			−1
*ND3*	3985	4348	364	+	ATG	T	32
*tRNA-Leu2*	4295	4371	77	−			−54
*tRNA-Asn*	4366	4419	54	+			−6
*tRNA-Ala*	4399	4455	57	+			−21
*tRNA-Ser1*	4438	4495	58	+			−18
*tRNA-Arg*	4496	4548	53	+			0
*tRNA-Glu*	4540	4598	59	+			−9
*tRNA-Phe*	4564	4616	53	−			−35
*ND5*	4631	6235	1605	−	ATT	TAA	14
*tRNA-His*	6263	6313	51	−			27
*ND4*	6294	7604	1311	−	ATA	TAA	−20
*ND4L*	7608	7896	289	−	ATG	T	3
*tRNA-Pro*	7854	7916	63	−			−43
*ND6*	7933	8337	405	+	ATA	TAA	16
*Cytb*	8345	9475	1131	+	ATT	TAG	7
*tRNA-Ser2*	9466	9528	63	+			−10
*tRNA-Thr*	9528	9588	61	+			−1
*ND1*	9566	10,477	912	−	ATG	TAA	−23
*tRNA-Leu1*	10,470	10,532	63	−			−8
*16S rRNA*	10,514	11,521	1008	−			−19
*tRNA-Val*	11,496	11,545	50	−			−26
*12S rRNA*	11,545	12,196	652	−			−1
*tRNA-Ile*	12,197	12,269	73	−			0
*tRNA-Gln*	12,271	12,322	52	−			1
CR	12,323	12,675	353	/			0
*tRNA-Met*	12,676	12,739	64	+			0
*ND2*	12,722	13,721	1000	+	ATT	T	−18
*tRNA-Trp*	13,657	13,718	62	+			−65
*tRNA-Tyr*	13,689	13,743	55	−			−30
*tRNA-Cys*	13,743	14	48	−			−1

**Table 3:** Composition and skewness of *Phormictopus cancerides* mitogenome.

Region	Size (bp)	A (%)	T (%)	G (%)	C (%)	A + T (%)	G + C (%)	AT-Skew	GC-Skew
Total genome	13,776	27.47	37.80	24.32	10.40	65.27	34.72	−0.158	0.401
PCGs	10,782	26.28	38.32	25.38	10.03	64.60	35.41	−0.186	0.433
tRNAs	1290	33.41	35.04	20.85	10.70	68.45	31.55	−0.024	0.322
rRNAs	1660	31.27	36.99	18.31	13.43	68.26	31.74	−0.084	0.154
CR	353	26.49	35.41	20.68	14.45	61.90	35.13	−0.144	0.177

## Data Availability

DNA sequences: GenBank accession number PV072888.
